# Higher throughput workflow with sensitive, reliable and automatic quantification of myelination in vitro suitable for drug screening

**DOI:** 10.1038/s41598-023-29333-1

**Published:** 2023-02-18

**Authors:** Sybille Seiler, Ciril Marius Wälti, Vanessa de Barros, Shahar Barbash, Lynette C. Foo

**Affiliations:** 1 pRED, Neuroscience, Discovery and Translational Area (NRD), F. Hoffmann-La Roche, Grenzacherstrasse 124, 4070 Basel, Switzerland; 2 Biozentrum, University of Basel, Spitalstrasse 41, 4056 Basel, Switzerland; 3 Quantified Biology, Cornell Tech, New York, NY 10044 USA

**Keywords:** Multiple sclerosis, High-throughput screening, High-throughput screening, Multiple sclerosis

## Abstract

Multiple sclerosis (MS) is the most common demyelinating autoimmune disease of the central nervous system (CNS). Immune-mediated myelin and axonal damage that is accompanied by chronic axonal loss causing destruction of the myelin sheaths are hallmarks of MS. While great strides have been made in understanding the molecular underpinnings of re-/myelination, currently no remyelination therapy is available for MS. As myelination is a complex process that is not fully understood, we sought to develop a systematic, reliable, automated and quantitative higher throughput screening method. We aimed to quantitate myelin sheaths in vitro with high sensitivity at the single cell level suitable for testing small compound libraries. To this end, we miniaturised in vitro retinal ganglion cell-oligodendrocyte precursor cell (RGC–OPC) co-cultures into a multi-well plate format. This allowed us to maintain the reciprocal interaction of live axons and oligodendrocytes (OLs) to ensure compact myelin formation. To quantify our co-cultures, we developed a novel computer vision algorithm to precisely measure myelination. We demonstrated efficacy of our system with known pro-differentiating compounds BQ3020 and XAV939 which exhibited robust, efficient, and dose dependent effects on myelination. Through this combination of experimental and technical advances, we have developed a method allowing systematic and reliable testing of remyelinating compound efficacy.

## Introduction

Myelin is a lipid-rich insulation sheath produced by OLs in the CNS. Myelin allows fast nerve transmission, protects axons and provides metabolic support to neurons^1^. A pathological feature of MS is myelin damage accompanied with axonal degeneration. Myelin dysregulation is not confined only to MS but has also been observed in many other neurodegenerative disorders^2–5^. Current standard-of-care of MS therapies ameliorate the autoimmune response accountable for destroying the myelin sheath that ultimately leads to the degeneration of the denuded axons. While these treatments effectively reduce relapses during the early stages of the disease, they are unable to halt disease progression very likely because they do not target remyelination directly^6,7^. One of the main bottlenecks of developing therapeutics to restore myelination is the lack of a systematic, reliable, automated and quantitative high-throughput screening platform to quantitate myelin sheaths in native systems consisting of functional, dynamic interactions between live axons and OLs in an unbiased manner. The reason for this is that myelination is a complicated, multicellular and multi-step process. Firstly, the OL precursor cells (OPCs) need to sense the demyelination and migrate to the site of the lesion where they divide and/or differentiate into myelinating OLs. They generate large amounts of myelin which then makes contact with an axon and ensheathes it^8,9^. As the myelin wraps around the axon, more and more is added to the inner tongue of the sheath and in the final stages as the cytoplasm is extruded from the sheath back into the OL cell body, myelin is compacted to form tightly packed layers. This is accompanied by the formation of paranodal junctions and the Nodes of Ranvier (NOR) along the axon. The NOR assembly is a high-density protein structure including clustered sodium channels. Clustering is initiated and supported by glial extracellular matrix (ECM) and axonal cell adhesion molecules (CAMs)^10–12^. To model these steps of myelination in vitro, multiple approaches have been used so far and all of them come with certain advantages and disadvantages. The simplicity of purified OPC monocultures, allowed the elucidation of the molecular mechanisms underlying OPC migration, proliferation and differentiation as well as stage-specific markers of the OL lineage (reviewed by^13–15^). Using such stage-specific markers, one can identify pro-differentiating compounds that are possible stimulators of remyelination^14,16–18^. A neuron-free model has been established to culture OPCs on nanofibres allowing the study of OPC-intrinsic mechanisms and fibre size or stiffness^19,20^. Myelination has been successfully achieved in these cultures. However, it has been shown that communication from axons regulate OL numbers, maturation, and survival^21,22^ via secreted factors^23–26^, contact-mediated signals^27–29^ and electrical activity^30,31^, which these OPC-nanofibre cultures do not provide. Various rodent co-culture systems providing OPC-neuron interactions have been established so far, including embryonic cortical cultures, co-cultures of purified cells^32,33^ and compartmentalized co-cultures using microfluidic devices^34^. They allow the examination of extrinsic mechanisms controlling myelination and are all suitable for relatively high-throughput compound screening. More physiological systems such as ex vivo slice cultures^35^, 3D brain organoids^36–38^ and human induced pluripotent stem cells (iPSC) derived co-cultures allow to examine demyelination and remyelination upon injury as well as genetically-related demyelinating disorders. However, their complexity, batch-to-batch variability, and intensity of labour as well as long cultivating periods make them unsuitable for high-throughput drug screenings^20^. Another disadvantage of these more complicated systems is that they often need to be cultured in the presence of serum, making the identification of single myelination regulators very challenging.

In contrast, co-cultures of purified primary re-aggregated retinal ganglion cells (RGCs) and cortical OPCs, cultured in a defined, serum-free media, are simpler but still provide sufficient complexity and the reciprocal communication between axons and OLs to examine all steps of myelination. A dense bed of axons forms within 2 weeks and they permit not only the study of all intrinsic but also extrinsic regulators of OL maturation and myelination by fluorescent microscopy imaging^32^. To obtain a more rapid high-throughput workflow that enables dose response testing of compounds in an unbiased manner, we combined and slightly adapted several existing procedures by miniaturisation of the RGC–OPC co-cultures into a multi-well plate format.

We have developed an in vitro workflow to test small molecule libraries for remyelinating drugs and focused on sensitivity to obtain dose response curves for these compounds. We developed several image analysis workflows using Perkin Elmer’s Harmony software and compared them to a novel unbiased, automated image acquisition module and a computer vision algorithm for the quantification of myelinating segments that we have developed. We applied known compounds with remyelination capacity such as the endothelin receptor agonist BQ3020^39^ and the tankyrase inhibitor XAV939^40^ to test and validate the sensitivity of our workflow.

In summary, we have used the OPC differentiation experiment to predefine the working dose range of pro-differentiating compounds and provide step-by-step instructions on how a precise in vitro readout for myelination at the single cell level was optimised. We found that our novel algorithm was sensitive enough to detect dose-dependent changes in response to remyelinating compounds. Taken together, we describe a novel system, combining experimental and computational elements, for a higher throughput in vitro screen of myelin-affecting compounds.

## Methods

### Animals

All CNS cells were purified from postnatal day P5-7 Sprague Dawley rats. All studies involving animals were carried out on the animal experimentation license BS-2931. The license was internally reviewed by our Animal Welfare Officer and submitted for approval to the authorities. There it was reviewed by the Ethical Committee on Animal Experimentation of Basel-Stadt and subsequently approved by the Cantonal Veterinary Office Basel-Stadt and the FSVO (Federal Food Safety and Veterinary Office). In addition, all in-vivo studies are reported in accordance with the ARRIVE guidelines for reporting experiments involving animals^41^. All animal care and experimental procedures were in accordance with national and international guidelines for animal care and were conducted according to the study protocol. The F. Hoffmann-La Roche Pharma Research Basel test facility is fully accredited by the Association for Assessment and Accreditation of Laboratory Animal Care International.

### OPC isolation

OPCs were isolated by immunopanning from P5-7 rat cortices as previously described^14^. Briefly, cortices were first enzymatically and then mechanically dissociated resulting in a single-cell suspension that was passed over a series of negative immunopanning plates to remove microglia, macrophages, endothelial cells (BSL1; Vector Labs L-1100, anti-CD45; BD Biosciences 550539) and mature OL precursors (anti-O1; MAB1327) before positively selecting for OPCs by an O4-coated (MAB1326) panning plate.

### OPC differentiation

The day before the cell preparation, the plate was coated with PDL-borate (poly-d-lysine diluted in borate buffer; Sigma P6407), washed three times with distilled water and dried overnight. The OPCs were cultured in serum-free media (OPC growth media) containing 100% DMEM (Gibco 11960-044), SATO (100 μg/ml transferrin; Sigma T-1147, 100 μg/ml BSA; Sigma A4161, 16 μg/ml putrescine; Sigma P5780, 60 ng/ml progesterone; Sigma P8783, 40 ng/ml sodium selenite; Sigma S5261), 100 U/ml penicillin, 100 μg/ml streptomycin, 110 μg/ml sodium pyruvate, 292 μg/ml l-glutamine, 5 μg/ml of N-acetylcysteine, 5 μg/ml insulin, 1000 × Trace Elements B (Cellgro 99-175-CI), 10 μg/ml d-Biotin and 2666 × vitamin B27 (Supplementary Table 1). Following growth factors were added: 10 ng/ml human platelet derived growth factor (PDGF; Peprotech 100-13-A), 10 ng/ml ciliary neurotrophic factor (CNTF; Peprotech 450-13), 10 ng/ml neurotrophin 3 (NT3; Peprotech 450-03) and 4.2 μg/ml forskolin (Sigma F6886). The cells were plated in a 96-well plate at the density of 5000 cells/µl by pipetting a droplet of 2 µl (containing 2500cells per µl) into the middle of the well and incubated at 37 °C for 5 min to allow the cells to settle. OPC growth media was added (200 µl/well) and cells recovered for 24 h. The OPC growth media was replaced by an OPC base media (without PDGF and Triiodothyronine (T3)) including the compounds BQ3020 (5–500 ng/ml) and XAV939 (50–5000 ng/ml) and the appropriate control, 0.1% DMSO. As a negative and positive control for OPC differentiation 10 ng/ml PDGF and 40 ng/ml T3 were used respectively. Cells were fixed 4 days (4d) post treatment. Compounds, tested for differentiation ability, were added in the absence of exogenous T3 as we have found that the addition of T3 itself masks effects of the compounds.

### RGC isolation

P7 RGCs were purified by immunopanning as previously described^42^. An additional anti-CD140b (BD Bioscience 558820) panning plate, to remove fibroblasts before positively selecting RGCs by a Thy1.1-coated (Thermo Fisher 15-0900-82) panning plate, was added.

### RGC-aggregate formation

RGC re-aggregates were made according to the protocol previously described^32^ with the modification of miniaturisation into a 96-well plate format to permit higher throughput imaging and quantification. Isolated RGCs were cultured in a defined serum-free base medium (ND-G) containing 50% neurobasal (Gibco 21103-049), 50% DMEM, 100 U/ml penicillin, 100 μg/ml streptomycin, 110 μg/ml sodium pyruvate, 292 μg/ml l-glutamine, SATO (100 μg/ml transferrin, 100 μg/ml BSA, 16 μg/ml putrescine, 60 ng/ml progesterone, 40 ng/ml sodium selenite), 5 μg/ml of N-acetylcysteine, 5 μg/ml insulin, 1000 × Trace Elements B, 10 μg/ml d-Biotin, 40 ng/ml T3 and 50 × vitamin B27 (Supplementary Table 1). This medium was supplemented with the following growth factors: CNTF at 10 ng/ml, brain derived neurotrophic factor (BDNF; Peprotech 450-02) at 50 ng/ml and forskolin at 4.2 μg/ml. Re-suspended RGCs were plated at 500,000 per well in an uncoated 96-well plate overnight at 37 °C. The RGCs were forced to form re-aggregates by pipetting up and down slowly and let them recover for 3 h at 37 °C. The RGC re-aggregates were then carefully re-suspended in the well and transferred to a 1.5 ml tube and the supernatant was checked for debris. ND-G media was added, after the re-aggregates had settled to wash and remove single cells. Washes were repeated until the supernatant was clear of debris and all cells transferred in ND-G media volume. All re-aggregates were plated at 100 µl per well in a PDL-borate/1 mg/ml laminin (R&D 3400-010-02) pre-coated 96-well plate. The cells were allowed to recover for 3 h in the incubator before 100 µl of ND-G media was added. RGC re-aggregates were cultured for 14 days and fed every 3 days with a half-change of ND-G media, of which the last feed contained no T3 (ND-G no T3) due to masking effect of exogenous T3 as explained in the OPC differentiation protocol.

### RGC–OPC co-cultures

One day before OPC addition, ND-G media in the 96-well plate was half-changed to serum-free base medium (Mym) containing 100% DMEM, 100 U/ml penicillin, 100 μg/ml streptomycin, 110 μg/ml sodium pyruvate, 292 μg/ml l-glutamine, 5 μg/ml insulin, 1000 × Trace Elements B, SATO (100 μg/ml transferrin, 100 μg/ml BSA, 16 μg/ml putrescine, 60 ng/ml progesterone, 40 ng/ml sodium selenite), 10 μg/ml d-Biotin, 10 mM hydrocortisone, 1 mg/ml ceruloplasmin, 1.36 mg/ml vitamin B12 (Sigma V6629), hormone mix (1 mg/ml apotransferrin, 20 mM putrescine, 4 µM progesterone, 6 µM sodium selenite) and 2666 × vitamin B27 (Supplementary Table 1) supplemented with CNTF at 0.01 ng/ml and BDNF at 50 ng/ml. A full change of Mym media with BQ3020 (1–500 ng/ml) and XAV939 (5–5000 ng/ml) was conducted before 5,000 OPCs/well were added. RGC–OPC co-cultures were treated again every 2-3 days with compounds in Mym media. Co-cultures were fixed 7 days post OPC addition.

### Immunohistochemistry

To perform immunohistochemistry, cells were washed once with dPBS and fixed with 4% paraformaldehyde in dPBS for 10 min at room temperature (RT). Cells were washed once with dPBS before adding blocking solution containing 50% goat serum, 50% antibody buffer (water, 150 mM sodium chloride, 50 mM Tris-base, 1% BSA, 100 mM l-lysine and 0.04% azide) and 0.4% Triton X-100 for 30 min at RT. Cells were incubated with primary antibodies overnight at 4 °C. The following day, the cells were washed with dPBS once and then incubated with secondary antibodies for 1 h at RT. The following primary antibodies were used: 1:100 rat anti-myelin basic protein (MBP) (MAB386), 1:1000 mouse anti-neurofilament H marker (Smi31) (Biolegend 801601) and visualised with appropriated secondary antibodies conjugated with Alexa fluorophores (Thermo Fisher) and DAPI at 1:1000.

### Imaging by Operetta/Opera Phenix

Perkin Elmer’s Harmony high-content analysis software 4.8 (HH17000001) was used to set up imaging settings for the 96-well plate. Imaging was done with a 20 × long WD non-confocal objective by the Operetta/Opera Phenix High Content Imaging System. Appropriate channels were selected (DAPI, Dyelight 647, Alexa 488) and exposure time as well as focus height was set accordingly. Imaging takes approximately 15 s/image. The images were either analysed directly by the Perkin Elmer’s Harmony software or exported as tiff files for analysis with Myelin Status.

### Sample preparation and gene expression analysis

RNA isolation from OPC/OL monocultures was performed using the miRNeasy Micro Kit (Qiagen 217084) according to the manufacturer's protocols using Qiazol lysis reagent. The RNA concentration and quality was measured using a Nanodrop system (Witec AG). The anchored-oligo(dT)^18^ Primer protocol of the Transcriptor First Strand cDNA Synthesis Kit (04897030001) was followed to reverse transcribe RNA. Real time quantitative PCR was performed using the LightCyclerR 480 SYBR Green I Master kit (04707516001) using the primer pairs for *Gapdh* (Fwd: *5′-CAA CTC CCT CAA GAT TGT CAG CAA-3′*, Rev: *5′-GGC ATG GAC TGT GGT CAT GA-3′*) and *Mbp* (Fwd: *5′-ACA CAA GAA CTA CCC ACT ACG G-3′*, Rev: *5′-GGG TGT ACG AGG TGT CAC AA-3′*). The following cycling conditions were applied for SYBR Green I mixtures; 20 μl sample volume, melting factor 1.20, 1 cycle pre-incubation at 95 °C for 600 s, 3 step amplification with 65 cycles at 95 °C for 10 s, 60 °C for 15 s, 72 °C for 15 s, 1 Melting cycle at 95 °C for 5 s, 55 °C for 30 s, 95 °C for 1 s, and 1 cooling cycle at 40 °C for 300 s.

### OPC differentiation analysis of OPC monocultures

We designed an analysis sequence by applying building blocks embedded in Perkin Elmer’s Harmony software based on counting the number of nuclei (DAPI^+^) and MBP^+^ cells. The building blocks are explained in detail for each sequence step and include image examples.

**Figure 1:**
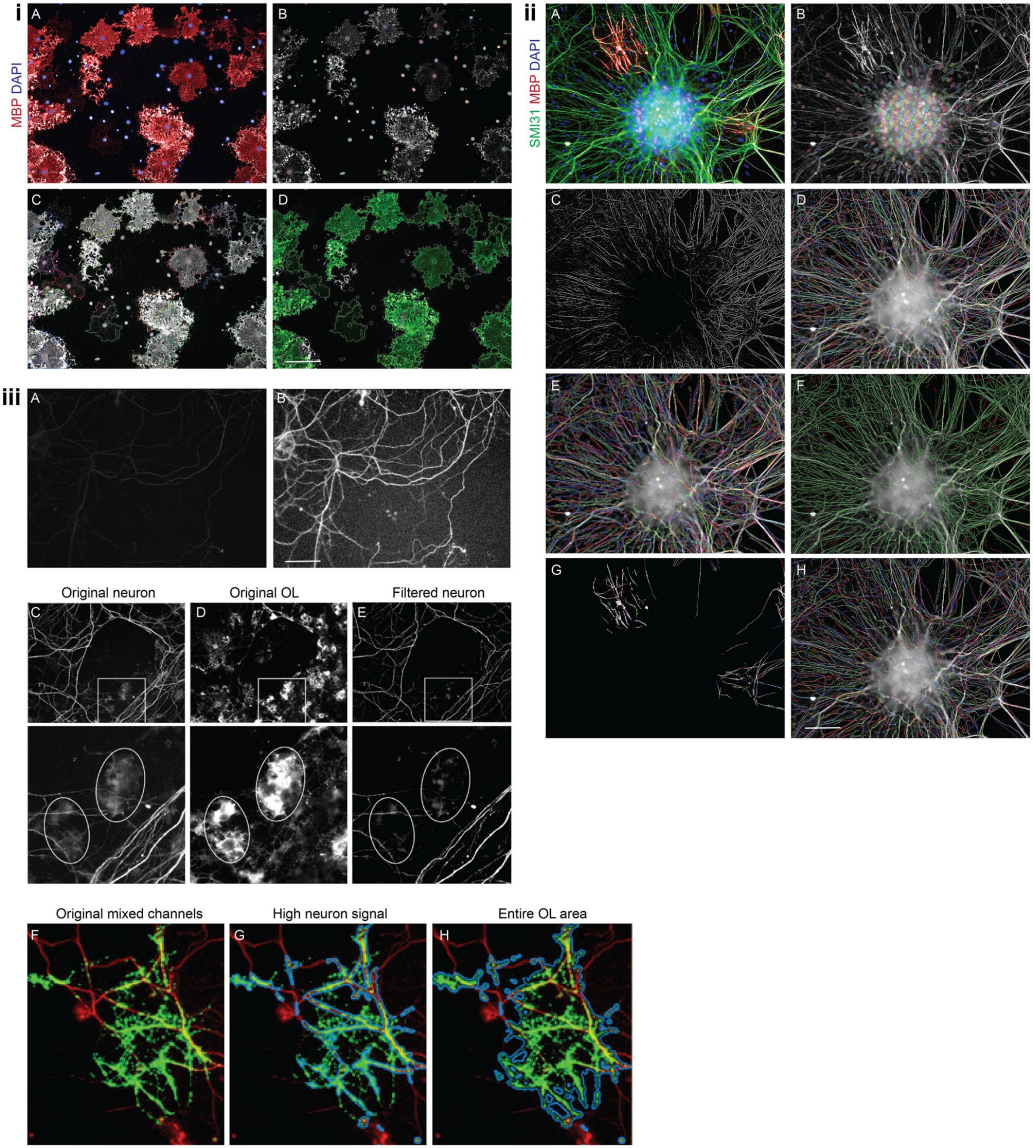
Representative images of OPC differentiation analysis sequence (**iA**) input data in step1, (**iB**) nuclei detection in step 2, (**iC**) cell cytoplasm detection in step 3, (**iD**) MBP+ population after definition of filter and characteristics properties in step 6. Representative images of length of myelinated segments calculation (**iiA**) input data in step 1, (**iiB**) nuclei detection in step 2, (**iiC**) image filtered by SER Ridge filter using Kernel normalization in step 3, (**iiD**) defining neurite segments by CSIRO neurite analysis in step 4, (**iiE**) background definition of neurite segment population in step 5, (**iiF**) axon selection from neurite segment population in step 10, (**iiG**) myelinated axon definition from axon population by Dyelight threshold settings in step 11, (**iiH**) unmyelinated axon definition in step 12. Representative images of myelin index calculation of RGC-OPC co-cultures (**iiiA**) raw neuron image and (**iiiB**) neuron image after contrast stretch in step 3, cleaning leak between channels (**iiiC**) original neuron (**iiiD**) original OL and (**iiiE**) filtered neuron in step 4, detection of OL area from (**iiiF**) original mixed channels the ratio of (**iiiG**) high neuron signal, calculated in step 6 and (**iiiH**) each entire OL area, calculated in step 5 represents the myelination index in step 8.

Step 1: The input data corresponds to microscope images from two channels; an OL-specific dye (Rat-MBP with Alexa Fluor 647) and a nuclei staining in blue (DAPI), for which flat field correction was done (Fig. 1iA).

Step 2: Cell nuclei are found via building block M that detects each nucleus as a region on the image having a higher intensity than its surrounding in the DAPI channel. It provides good results using following second level parameters: diameter, detection sensitivity, splitting sensitivity and common threshold from the DAPI channel (Fig. 1iB). Building block M is working well for similar sized nuclei, as this is an OPC monoculture.

Step 3: Cell cytoplasm is recognized by applying building block D that determines the intensity threshold for each object individually and uses a restrictive region. It selects the nuclei population defined in Step 2 as region of interest (ROI) in the Dyelight 647 channel from the MBP staining (FIg. 1iC). This is the best applicable in this case as intensity decreases with the distance from nucleus.

Step 4: The mean intensity of the cytoplasm’s are calculated.

Step 5. Morphology properties are calculated for Dyelight 647 channel to propose cytoplasm output that can be adjusted by changing the intensity, cytoplasm area (µm^2^) and additional characteristic as roundness, width, length or ratio of width (µm) to length (µm) of the cytoplasm manually.

Step 6: The MBP^+^ population is selected by applying a filter by property: in this case the mean intensity of Dyelight 647 (100–150) and cytoplasm area (200–300 µm^2^) revealing the MBP^+^ cells in green (Fig. 1iD).

Step 7: To define the results, a list of output involves the number of total nuclei, the number of MBP^+^ cells and properties of MBP^+^ population (mean intensity of Dyelight 647, cytoplasm area (µm^2^), cytoplasm roundness and amount of objects per field of view).

Step 8: To obtain the ratio of MBP^+^ cells (a) to the total cell number (b), a formula output a/b is added to the results definition. The normalized MBP^+^ cells are then compared across conditions.

### Calculating total length of myelinated segments of RGC–OPC co-cultures

We designed an analysis sequence in Perkin Elmer’s Harmony software to define the total length of myelinated segments of axons, a modified approach of quantifying myelination from a method previously described. They have defined myelination as total length of contiguous, aligned MBP staining normalized by total OL number (fibre length/number of Olig2^+^ nuclei)^43^. As one OL can myelinate multiple segments of an axon simultaneously, we only considered stretches of MBP overlapping with a neurofilament heavy subunit marker Smi31, rather than normalising MBP staining by the total number of OLs.

Step 1: The input data corresponds to microscope images from three channels; one for a neuron-specific green dye (here Ms-Smi31 visualized with Alexa Fluor 488), an OL-specific dye (here Rat-MBP visualized with Alexa Fluor 647) and a nuclei staining (DAPI) (Fig. 1iiA). No flatfield, but brightfield correction is selected and individual planes are processed.

Step 2: Cell nuclei are identified again via the building block M from the DAPI channel and the nucleus inner centre region is selected (Fig. 1iiB), meaning the nucleus inner centre is defined as the output region.

Step 3: The image is filtered by a SER Ridge filter at 1px scale and uses a Kernel normalization. The latter is an image-processing feature that is used for blurring, sharpening, embossing and edge detection and therefore the filtered images are pixel wise divided by the smoothed original image (Fig. 1iiC).

Step 4: ‘Neurite segments’ proceeding from the nuclei inner centre region are selected in the SER Ridge filtered channel by the building block ‘CSIRO neurite analysis’ (Fig. 1iiD). An algorithm, developed by the Australian CSIRO research institute that uses following adjustable parameters, does the analysis:

1. Smoothing (Gaussian Filtering) by selecting the level of Gaussian blur
2. Linear feature detection: the neurites are detected in this step by the tuning parameters linear window and contrast
3. Remove small objects by changing the diameter
4. Closing gaps between detected neurites

Step 5: The background of the selected neurite segments population is defined via resizing the region (µm/px) by tuning the second level parameter outer and inner border (in this case the outer border is defined as − 3px) (Fig. 1iiE). The unit of the parameters is an absolute distance, either micrometre (µm) or pixel (px). The ‘keep image border’ option defines the behaviour when the original region touches the image border and in this case the option is activated meaning the pixels at the image border are not modified. That corresponds to the assumption that the object border coincides with the image border.

Step 6: Mean intensity properties are calculated to define Dyelight 647 intensity of ‘Neurite Segments’ and distinguish from background.

Step 7: To calculate the Dyelight 647 difference (MBP staining); the mean background intensity (= B) (defined in step 6) is subtracted from the mean intensity of ‘Neurite Segments’ (= A) by formula method (A–B).

Step 8: The mean intensity of the ‘Neurite Segment’ population in the Alexa 488 channel is calculated.

Step 9: From the ‘Neurite Segment’ population the axons are selected via common filter that removes boarder objects.

Step 10: ‘Axons’ with manual adjusted intensity are selected from the ‘Neurite segment’ population (Fig. 1iiF).

Step 11: To define myelinated axons from the ‘Axon’ population; the Dyelight thresholding is set manually (by optimizing Dyelight difference for example > 100) (Fig. 1iiG).

Step 12: Unmyelinated axons are selected as all axons with the requirement myelinated axons = 0 which were defined in Step 10 (Fig. 1iiH).

Step 13: Image analysis results are calculated by summing ‘myelinated axons’—segment length (µm) per well.

Step 14: Total length of ‘myelinated axons’ per well (µm) is normalized to its respective control and compared across conditions.

### Calculating myelination index of RGC–OPC co-cultures

We developed the following algorithm to estimate myelination status of individual cells, i.e. myelination index. The sequence of steps are explained here and the detailed algorithm is available via GitHub link.

Step 1: The input to the algorithm is composed of microscope images from two channels; one for a neuron-specific dye and another for OL-specific dye.

Step 2: The general noise of each image (neuron and OL separately) is measured with ‘salt and pepper’ noise estimation. This allows for filtering-out of noisy images when analysing an image set.

Step 3: Contrast stretch of intensity values is performed for each image, to bring the general intensity distributions across images closer together. The stretch saturates the highest and lowest 1% pixels in each images. Figure 1iiiA is an example of a raw neuron image and 3B after contrast stretch.

Step 4: Cleaning leak between channels.

- Leak from neuron channel into the OL channel introduces linear objects that are far away from the cell body and are likely not part of an OL. To filter those out, the algorithm performs a wide Gaussian filtration, with size of the averaged OL, on the OL image and increases intensity levels for OL image proportionally to the filtration output.
- Leak from OL channel into neuron channel introduces non-linear, ‘blobby’ objects in the neuron image that are likely not part of a neuron cell. To filter those out, the algorithm performs a wide Gaussian filtration, with size of the averaged OL cell, on the neuron image and decreases intensity levels for the neuron image proportionally to the filtration output.
- If leak is too high, based on local spatial correlation between channels, the algorithm excludes the image.

Figure 1 iiiC–E is an example of neuron channel cleaned from OL channel leak. Shown are full imaging field and zoom-in for the area marked by the white square. The fuzzy, blobby signals in the neuron channel (Fig. 1iiiC) enclosed in the marked ellipses likely represent leakage from the OL channel (Fig. 1iiiD). Note that after filtration as described above; those blobs are less prominent compared with the linear structures representing neuronal projections (Fig. 1iiiE).

Step 5: Detection of OLs. The algorithm takes the 20–80% middle range of intensity after the corrections described above. In this range, the algorithm examines the binary objects above a series of intensity thresholds. Only objects above a certain size are considered, specifically 20% of the averaged OL cell in the experiment. The algorithm then selects the intensity threshold in which the highest number of cells were detected. Areas above this intensity threshold are defined as detected OL cells.

Step 6: Inside the regions of cells detected in the last step the algorithm finds sub-regions of high intensity of neuron signal. Intensity threshold for neuron signal is determined by the highest 80% (i.e. above 20%) after the corrections described above.

Step7: Excluding two types of artefacts.

- Neuron aggregates have very high local intensity level that obscures cellular details. These regions are identified with image-erosion with a circle of a diameter of 60 pixels.
- Dust specks are seen on images as bright circle objects. In the algorithm circularity of each object is measured and objects with circularity above 0.9, which are likely dust particles, are excluded.

Step 8: For each OL cell (Fig. 1iiiF) the ratio between the areas of high neuron signal (Fig. 1iiiG, calculated at step 6) and total cell area (Fig. 1iiiH, calculated at step 5) is calculated. This ratio is the myelination index.

To compare myelination index with manual scoring, we randomly shuffled experiment images and asked an expert user to score myelination on a one to five scale. We then calculated Pearson correlation coefficients for manual and algorithm`s scores, per data set (see results in Fig. 6).

### Statistical analysis

GraphPad Prism 8.4.2 software and Myelin Status were used to do all statistical analyses. Data are presented as mean ± SEM., unless otherwise indicated. Statistical analyses were conducted using the Student’s T-test, the one-way ANOVA with Dunett’s multiple post-hoc tests or non-parametric tests, such as the Mann–Whitney U-test, when data was not normally distributed. Details on the type of test applied for each experiments are mentioned in the figure legends and results section. In general, n values refer to the number of individual dots, differentiations, or imaging fields for a given experiment; specifics are provided for each experiment in the corresponding figure legend.

## Results

We established a new higher throughput workflow including optimised cell-culture systems, imaging method and automated analysis to assess myelinating affecting compound efficacy. Immunopanning was used to isolate primary rat OPCs and RGCs. Primary OPC monocultures and RGC–OPC co-cultures were miniaturised and set up for automated imaging with Operetta/Opera Phenix from Perkin Elmer. Obtained images were used to quantify OL differentiation, myelin sheath length and myelin status at the single cell level. For latter, a novel myelin status algorithm was developed for sensitive quantification of myelination (Supplementary Fig. 1).

### Optimising OPC to OL differentiation

As a first step, we have optimised our culturing, imaging and analysis techniques for the first stage of myelination, namely OPC to OL differentiation for drug discovery purposes. To illustrate this, we have used several compounds known to induce OPC to OL differentiation. Primary rat O4^+^ OL precursor cells were stimulated with increasing T3 concentrations (0.4–200,000 ng/ml), BQ3020 (5–500 ng/ml) and XAV939 (50–5000 ng/ml) for 4 days in absence of PDGF.

**Figure 2:**
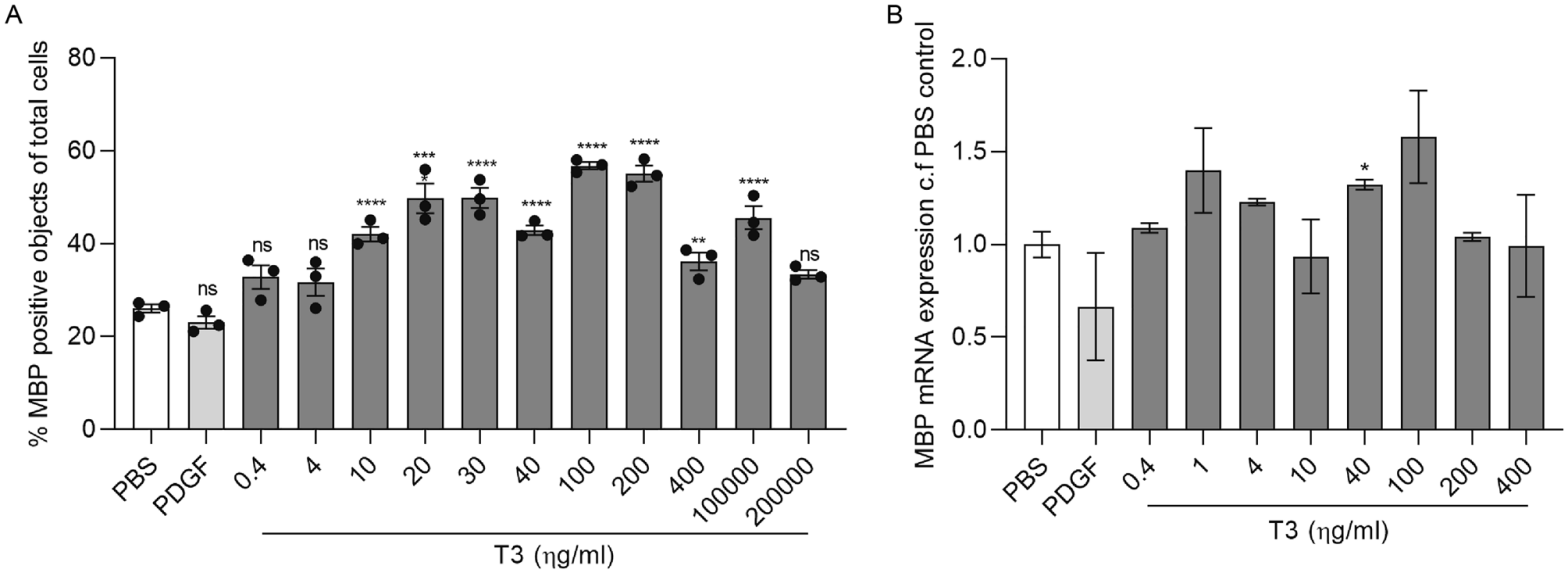
T3 promotes OPC into OL differentiation. (**A**) Differentiated OLs quantified from MBP^+^ stained cells normalized to total cell number. Four day treatments of O4^+^ OPCs with increasing concentrations of T3 (0.4–200,000 ng/ml), n = 3 are compared to PBS control and PDGF negative control. (**B**) Fold change MBP mRNA expression of differentiated OLs with increasing concentrations of T3 (0.4–400 ng/ml), n = 2 are compared to PBS control and PDGF negative control. Individual values show mean ± SEM, analysis was done by an unpaired t-test with 95% confidence interval, *ns* not significant, *P < 0.05, **P < 0.01, ***P < 0.001, ****P < 0.0001.

We designed an automated analysis sequence using building blocks in Perkin Elmer’s Harmony high-content analysis software based on the number of nuclei (DAPI^+^) and MBP^+^ cells resulting in a precise ratio of differentiated OLs. Comparing myelin basic protein positive (MBP^+^) cells normalized to total cell number (DAPI^+^), a significant increase in differentiated OLs was observed with increasing T3 concentrations starting from 10 ng/ml. We provide the effective dose curve (ng/ml) of T3 on OL differentiation in Fig. 2. As a positive control for OPC differentiation, the T3 concentration of 40 ng/ml was selected as it robustly and significantly increased OPC differentiation to 40% MBP^+^ cells after 4 days across experiments (Fig. 2A), as well as significantly showed increased MBP mRNA expression compared to PBS control (Fig. 2B). To lower the baseline for identifying candidate compounds, T3, which tends to mask potential pro-myelinating stimulators, was eliminated in all further treatment conditions. T3 (40 ng/ml) and PDGF (10 ng/ml), an OPC mitogen, were used as positive and negative controls respectively.

**Figure 3:**
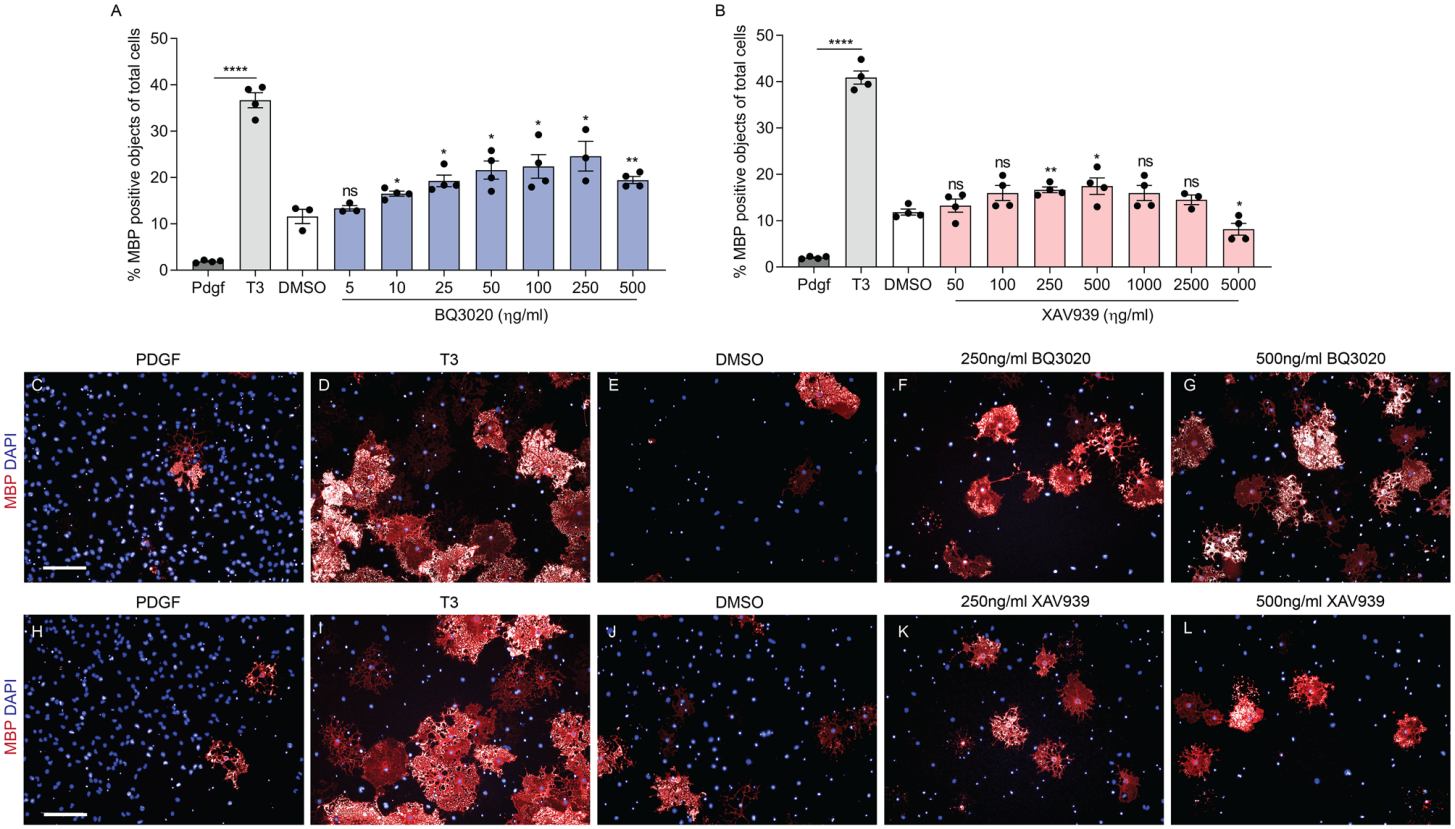
BQ3020 and XAV939 promote OPC differentiation in vitro*.* Differentiated OLs quantified by MBP^+^ stained cells (%) normalized to total cell number. Four-day treatments of O4^+^ OPCs with increasing concentrations of (**A**) BQ3020 (5–500 ng/ml) and (**B**) XAV939 (25–5000 ng/ml) were compared to DMSO control. (**C**,**H**) PDGF (10 ng/ml) and (**D**,**I**) T3 (40 ng/ml) are displayed and quantified as negative and positive controls, respectively. Representative immunofluorescent images of OPC/OLs monoculture highlighting nuclei in blue (DAPI) and differentiated cells in red (MBP) in (**E**,**J**) DMSO control condition and (**F**,**K**) 250 ng/ml BQ3020 and XAV939 and (**G**,**L**) 500 ng/ml BQ3020 and XAV939 conditions respectively (scale bar 100 µm). Individual values show mean ± SEM; 3–4 biological replicates were done, analysis was done by an unpaired t-test with 95% confidence interval, *ns* not significant, *P < 0.05, **P < 0.01, ***P < 0.001, ****P < 0.0001.

BQ3020 showed an increasing OL differentiation promoting effect from 10 ng/ml onwards and revealed its maximal effect at 250 ng/ml compared to DMSO (Fig. 3A). Similarly, a dose response curve was observed with increasing concentrations of XAV939 (50–5000 ng/ml), as well as a maximal effect at 250–500 ng/ml XAV939, compared to DMSO. The highest proliferation and minimal differentiation of OPCs was observed as expected, with PDGF (Fig. 3C,H). The highest amount of differentiated cells were clearly visible by the numerous MBP sheaths in addition of T3 (Fig. 3D,I). The significantly increased number of differentiated OLs upon BQ3020 (Fig. 3F,G) and XAV939 (Fig. 3K,L) administration compared to the DMSO controls (Fig. 3E,J) is visible from the MBP sheath to DAPI ratio.

### Quantifying myelination in co-cultures

We also sought to develop a method to reliably quantify myelination such that dose response curves in response to remyelinating compounds (BQ3020, XAV939) can be obtained. We have compared and contrasted two methods to quantify myelination in co-cultures; one evaluates the total length of myelinated segments and the second takes into account the status of individual processes of an OL to determine its ‘myelin status’. We have found that the myelin status gives a more sensitive readout, enabling us to obtain dose–response curves suitable for determining efficacy of compounds that promote myelination. While MBP was used as a marker for OPC differentiation and myelin formation, a mouse anti-Smi31 antibody was used to stain the neurofilament heavy polypeptide (NF-H), intermediate filaments found in neurons. This antibody was selected as axonal marker of the retinal ganglion cells throughout the study.

We observed that a dense bed of axons formed within 12–14 days after seeding of the neurons. Upon OPC addition, successful differentiation into OLs, tracked by the production of MBP sheaths, was observed and we then proceeded to quantify myelination in response to previously published compounds. The predefined dose range of BQ3020 and XAV939 was added to the RGC–OPC co-cultures for 7 days.

**Figure 4:**
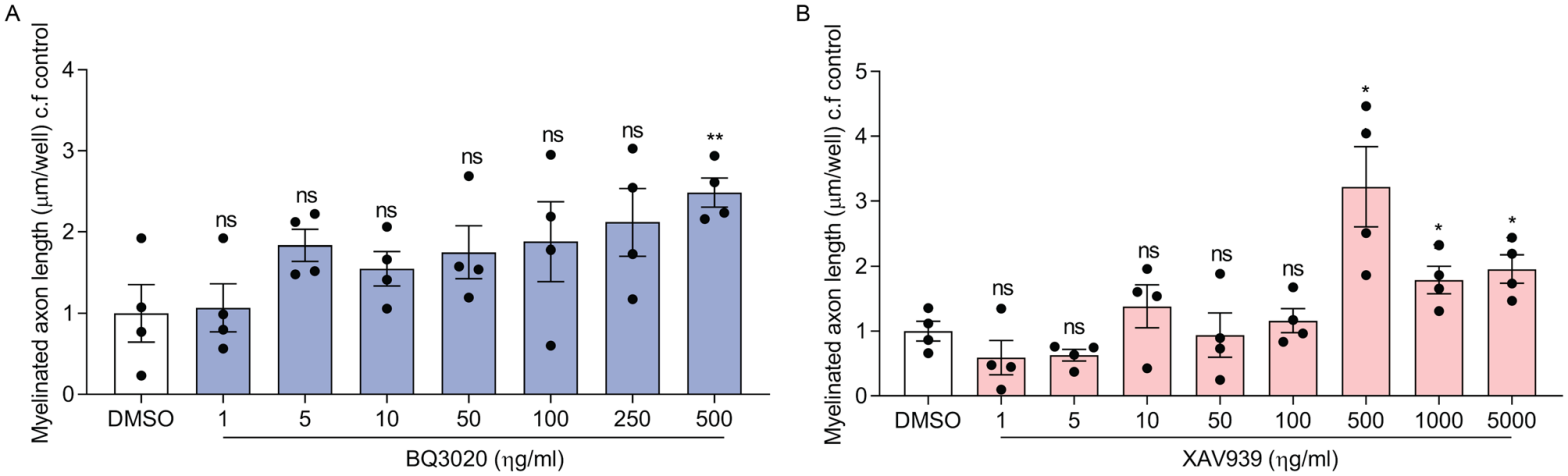
Higher concentrations of BQ3020 and XAV939 increase the total length of myelinated axons in vitro*.* Quantification of total myelinated axon length (µm/well) of (**A**) BQ3020 (1–500 ng/ml) and (**B**) XAV939 (1–5000 ng/ml) compared to DMSO control. Individual values show mean ± SEM; 4 biological replicates were done, analysis was done by unpaired t-test with 95% confidence interval, *ns* not significant, *P < 0.05, **P < 0.01, ***P < 0.001, ****P < 0.0001.

In our first analysis method, we generated an analysis sequence using building blocks in Perkin Elmer’s Harmony high-content analysis software. The analysis was designed to measure the total length of myelinated segments in the co-cultures by recognizing the MBP sheath of OLs overlapping with the axons. Previously, the total length of myelinated axons had been defined as total length of contiguous, aligned MBP staining normalized by total number of OLs (fibre length/number of Olig2^+^ nuclei) by Lariosa-Willingham et al.^43^. However, we specified the analysis by counting only the continuous stretches of MBP overlapping with axonal Smi31 staining. This fibre length calculation added all ensheathed and myelinated axon segments per experimental area (µm/well). The total length of myelinated axons slightly increased with increasing concentrations of BQ3020 (1–500 ng/ml) compared to DMSO control, although the variance within the conditions was relatively high. The maximal length of myelinated axons (µm/well) and only significant effect on the myelin sheaths was observed with 500 ng/ml BQ3020 (Fig. 4A). Similarly, high variability across replicates and only a significant difference in total myelinated axon length (µm/well) with higher concentrations of XAV939 (500–5000 ng/ml) was observed compared to DMSO control (Fig. 4B). Together, BQ3020 and XAV939 did not reveal a clear dose-dependent effect on the myelinated axon lengths.

**Figure 5:**
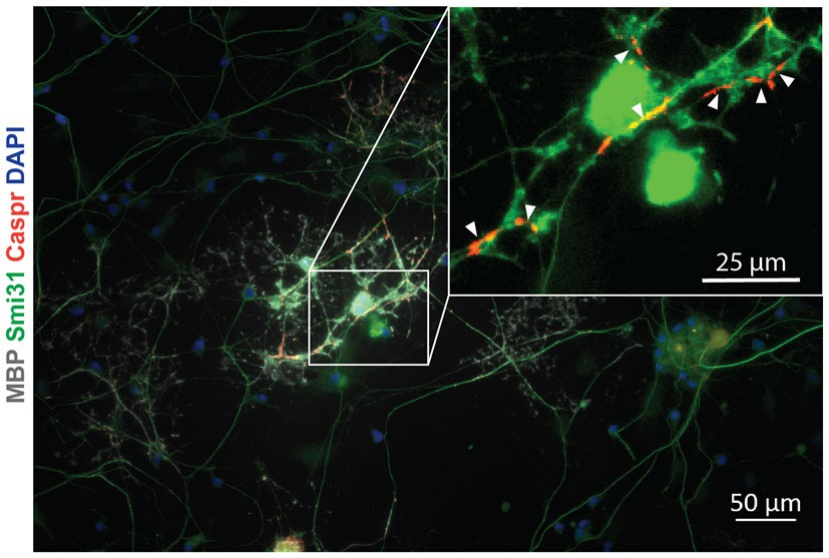
Compact myelin formation occurs in 96-well RGC–OPC co-cultures in vitro*.* RGC–OPC co-culture in a 96-well plate format stained for MBP (grey), Smi31 (green), Caspr (red), and DAPI (blue). White arrows indicate paranodal Caspr staining (red) overlapping with Smi31 (green) and therapy flanking the NOR.

To determine if we were able to form compact myelin in culture, we examined the NOR formation in our cultures. NORs consist of para- and juxaparanodal structures that are essential to the action potential propagation in the CNS. As an indicator of early node formation, and a surrogate for compact myelin formation, we found that prior to NOR formation, the paranodal Contactin-associated protein (Caspr) has a more diffuse localisation along the length of the axon. However, upon formation of a myelin segment, Caspr redistributes and becomes concentrated in the paranodal junction of mature myelinated axons^28,44,45^. Figure 5 depicts the successful compact myelin formation by an OL, shown by Caspr (red) stained paranodes along the axon (green) enclosing the NOR.

**Figure 6:**
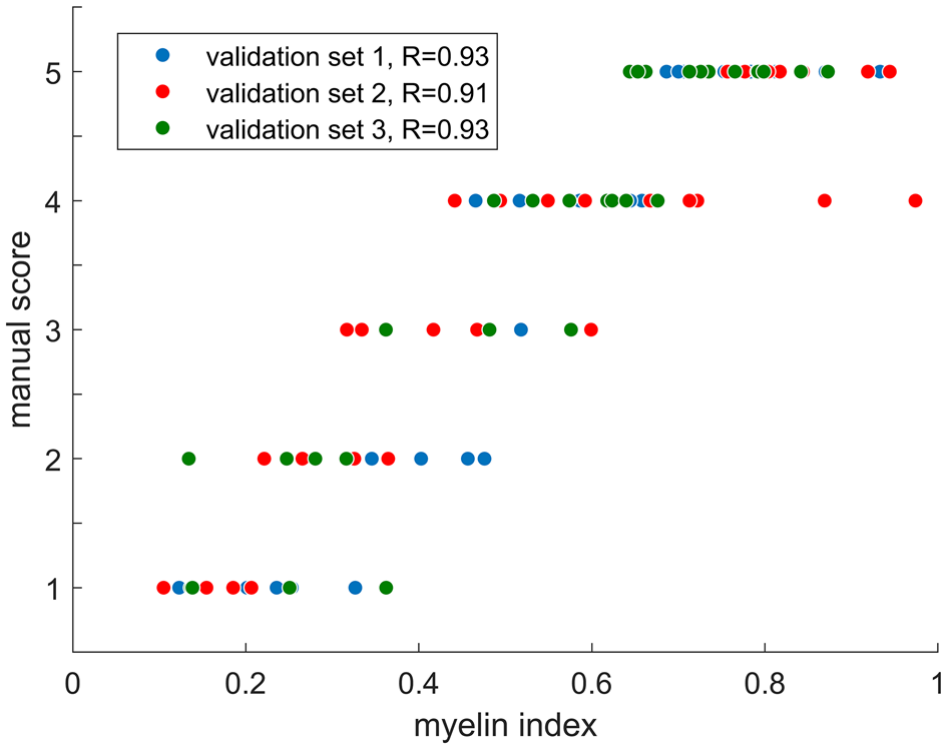
Comparison of myelination index with manual scoring. Shown are manual scoring, on a scale of 1–5, against myelination index values, across individual cells. Colors show datasets of different conditions and time. Also shown are Pearson correlation coefficients for each dataset.

**Figure 7:**
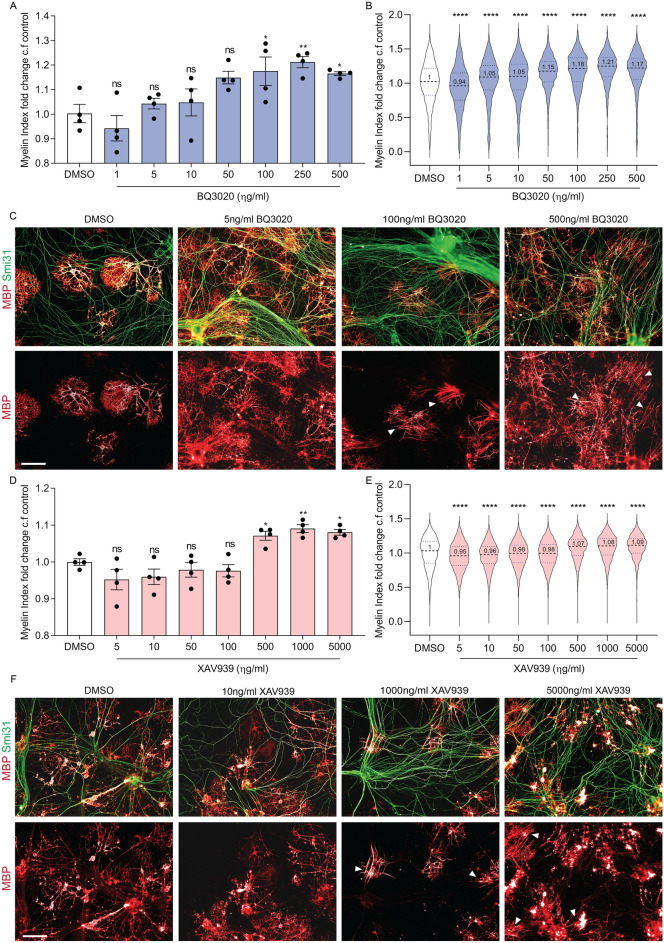
BQ3020 and XAV939 facilitate myelination in vitro*.* Quantification of myelination (myelination index) of RGC–OPC co-cultures treated with increasing concentrations of (**A**) BQ3020 (1–500 ng/ml) and (**C**) XAV939 (1–5000 ng/ml) for 7 days and normalized to DMSO control. Myelin index of the OL population per condition plotted as violin plot in (**B**) for BQ3020 treatments and (**E**) for XAV939 treatments normalized to DMSO control. Representative images of fluorescent MBP (red) and Smi31 (green) of C) BQ3020 and (**F**) XAV939 conditions compared to DMSO used for quantification (scale bar 100 µm). White arrows indicate axons wrapped with compact myelin by an adjacent OL. Individual values show mean ± SEM; Violin plots show median and quartiles as well as the mean as number, 4 biological replicates were done, analysis was done by one-way ANOVA followed by Dunnett’s multiple post hoc test for comparing more than three biological samples, *ns* not significant, *P < 0.05, **P < 0.01, ***P < 0.001, ****P < 0.0001.

After confirming that we could observe paranodal junction formation in our co-cultures by Caspr localization in the 96-well plate format, we focused on developing a more sensitive myelination quantification method. One that takes the morphology changes during OPC differentiation into a myelinating OL into account. OL lineage development can be traced by their morphological changes from a highly proliferative unipolar cell towards an elongated proliferative OPC differentiating into a polygonal promyelinating cell with ramified processes and finally a myelinating, non-proliferative cell with numerous parallel aligned process arbours that can myelinate multiple axons^46^. For this, we tailored a computer-vision algorithm to identify only the myelinating parts of an OL. This algorithm termed ‘Myelin Status’ was designed to mimic the way a researcher assesses myelination images by eye. The full, systematic algorithm is described in the methods section. Briefly, the algorithm detected individual OLs, mapped nearby neuronal projections, and calculated the ratio between the myelinating parts of an OL cell and its total area, while filtering out artefacts such as neuron-aggregates. The output was a score of the degree of myelination for each individual OL. The myelination index was validated against a human researcher. Specifically, three data-sets of varying experimental conditions and from three different plate batches were analyzed. In each one, 30 randomly selected cells were presented to a researcher. The researcher was asked to score them on a scale of 1 to 5, as minimally myelinating to maximally myelinating. Lastly, Pearson correlation coefficient was calculated between the ordinal (examiner score) and continuous (myelin index) variables in the three data-sets and was found to be 0.93, 0.93 and 0.91 (Fig. 6). In addition, we demonstrated that our algorithm was sensitive enough to reveal dose responses of (re-)myelinating compounds. After, quantifying the total myelinated fibre length, the image analyses of the RGC–OPC co-cultures treated with BQ3020 and XAV939 were repeated with Myelin Status. RGC–OPC co-cultures treated with BQ3020 (1–500 ng/ml) for 7 days facilitated myelination from 100 ng/ml onwards and revealed its maximal promotion at 250 ng/ml compared to DMSO control (Fig. 7A). Once the myelin index of each OL of the population of a condition was plotted, the significant difference of the OL myelination behaviour became even more prominent (Fig. 7B). Through the ability of the algorithm to analyse the entire experimental area (96-well plate) we receive an overview of the entire OL population within 9 min per well (one well containing 5,000 OPCs was imaged with approx. 69 images at a rate of 7.8 s/image). The morphology of ensheathing OLs was apparent in the DMSO, 5 ng/ml BQ3020 and 10 ng/ml XAV939 condition by its broad, ramified character, whereas myelinating OLs were marked by compact, linear stretches of MBP in the 100–500 ng/ml BQ3020 (Fig. 7C) and 1000–5000 ng/ml XAV939 (Fig. 7F) condition. The compound XAV939 facilitated myelination in RGC–OPC co-cultures significantly from 500 to 5000 ng/ml (Fig. 7D). Lower concentrations of XAV939 (1–100 ng/ml) showed that the majority of OL population were similarly or slightly less myelinating compared to DMSO control. Even though, 5000 ng/ml XAV939 revealed decreased OPC differentiation in the OPC monoculture, we observed a significant increase in myelination in the RGC–OPC co-cultures (Fig. 7D,E). In its current form, our assay workflow analyses the entire experimental area, circumventing any sampling bias and has allowed us to study and identify many early- and late-stage regulators of myelination.

By showing these sensitive readouts and quantification of myelination in vitro, we conceptualized the parameters, rules and models necessary to characterize a myelinating OL and its myelinating capacity in a programmable framework resolving in a myelination index. We provide step-by-step instructions on how we defined the readout of myelination in our system and demonstrated that our analysis is sensitive enough to show dose responses of remyelinating compounds, while at the same time is able to process large data sets that would simply overwhelm human analysis.

## Discussion

The emergence and amenability of higher throughput systems to study CNS myelination in native systems in vitro as well as their automated quantification is key to the search of more efficacious therapeutics for demyelinating diseases. The differentiation of OLs at the site of demyelinated lesions creates only one part to the solution of regaining myelination and proper nerve function. Therefore, for future drug development, we believe a simple and easy-to-manipulate myelin formation in vitro model that is still complex enough to assess later stages of myelination in an automated way provides all necessities to better understand regulators of myelination in health. By using the OPC differentiation assay and semi-automated analysis in a first step, the pro-differentiating effects of compounds as well as their working dose range can be rapidly assessed.

In a second step, the RGC–OPC co-culture system of purified cells that is miniaturised to a higher throughput setting with fully automated analysis provides insight into extrinsic processes and effects on later stages of myelination. Our workflow was based on previously established and highly efficient assays to provide robust, reproducible and automated results at the single cell level. Unlike other methods, our assays were made of unpassaged primary rodent cells in serum-free conditions to get closer to in vivo-like cellular phenotypes. Also, compound testing was performed in the absence of exogenous T3 to prevent the masking effect of T3 on OPC differentiation. In line with the results from Yuen et al.^39^, 50–100 ng/ml BQ3020 revealed a significant promoting effect on OPC differentiation, although our readout showed already a significant increase in differentiated OPCs from lower concentrations (10 ng/ml). We revealed the potent OPC differentiating dose-dependent effect and a peak at 250–500 ng/ml XAV939 in vitro, compared to Fancy et al.^40^, who presented effective XAV939 concentrations in vivo at a lower nanomolar range (3–30 ng/ml). After confirming the production of compact myelin by OL in our miniaturised native culture system, the same dose ranges of BQ3020 and XAV939 were used for the RGC–OPC co-cultures to quantify myelination first: by the length of total myelinated axons, secondly by the myelin status that examines single OPCs. Through the readout of the myelinated axon length per condition that was adapted from a previous method^43^, we did not observe a significant dose-dependent increase in myelinated axon lengths with increasing concentrations of BQ3020 and XAV939. Furthermore, the variance between replicates was relatively high, likely because the calculation of myelinated segment-lengths were based on the mean channel intensities. To the date, there is limited knowledge of how myelin lengths are established. In vivo, myelin sheath size often scales with the calibre of the axons that are being ensheathed, but the mechanism for how oligodendrocytes respond to axon diameter to control different myelin sheath lengths remains unknown. However, accumulating in vitro evidence supports an oligodendrocyte-driven, neuron-independent ability to differentiate and form initial sheaths depending on their regional identity^47^. Assuming that additional, still incompletely understood regulatory mechanisms are involved in defining the total length of a myelinated axons, makes this readout complex and less suitable to assess myelinating compound efficacy. Previous methods have normalized the total lengths of MBP stretches by the total number of OLs. However, this does not imply the necessary axon contact as well as assumes similar MBP protrusions per cell. In reality, both an intrinsic clock and extrinsic regulators stimulate OPC differentiation and maturation, meaning one cell can already be at the end-stage of differentiation while another can still have a bipolar non-myelinating morphology. To differentiate between these stages of OPC differentiation and maturation, for example by distinguishing an ensheathing from a myelinating OL, we scored each individual cell with a novel tailored computer-vision algorithm. The automatic identification, channel corrections and noise cancellation as well as taking a middle range of signal intensity of objects of certain sizes provided a more precise and robust readout and for the first time enough sensitivity to capture dose depending effects.

To overcome limitations like the lack of disease context as well as translatability to humans we hope to improve and adapt this automated analysis to more complex myelin formation systems. If automated confocal imaging of 3D organoids can be established and imaging made compatible with our algorithm, sensitive myelin index readouts could be possible at the single cell level without compromising on a higher throughput method. Furthermore, once an effective in vitro dose range of a compound is established with this workflow the predictions to in vivo dosing are simplified and saves overall time and resources. Altogether, the described platform using rodent co-cultures and automated analysis is easy to set-up, relatively inexpensive and builds a great tool to evaluate the dose-depending effect of candidate compounds or to study regulators of all steps of CNS myelination.

## Conclusion

Here, we have described two novel things: (1) the adaptation of a native primary cell co-culture system for relatively high-throughput drug screening with industry rigor and (2) a rapid computer-vision analysis method to reliably quantify myelination in vitro. We hope that other groups will find this workflow with paradigms relevant to the MS field advantageous for comprehensively examining future relevant promyelinating compounds.

## Supplementary Information

Supplementary Information.

## Data Availability

The datasets generated and/or analysed during the current study are not publicly available due as the thousands of raw images analysed are very large but will be made available from the corresponding authors on request.

## Code availability

The algorithm generated and used for analysis during the current study is available in the following repository (https://github.com/sbarbash/MyelinationStatus).
