# Supplementary material for: Higher throughput workflow with sensitive, reliable and automatic quantification of myelination in vitro suitable for drug screening
Source: Sci Rep. 2023 Feb 18;13:2883. doi: 10.1038/s41598-023-29333-1 (PMC9938858; doi:10.1038/s41598-023-29333-1)
Supplement: Supplementary file 1 — Supplementary Information. [file 41598_2023_29333_MOESM1_ESM.pdf]

Supplementary information

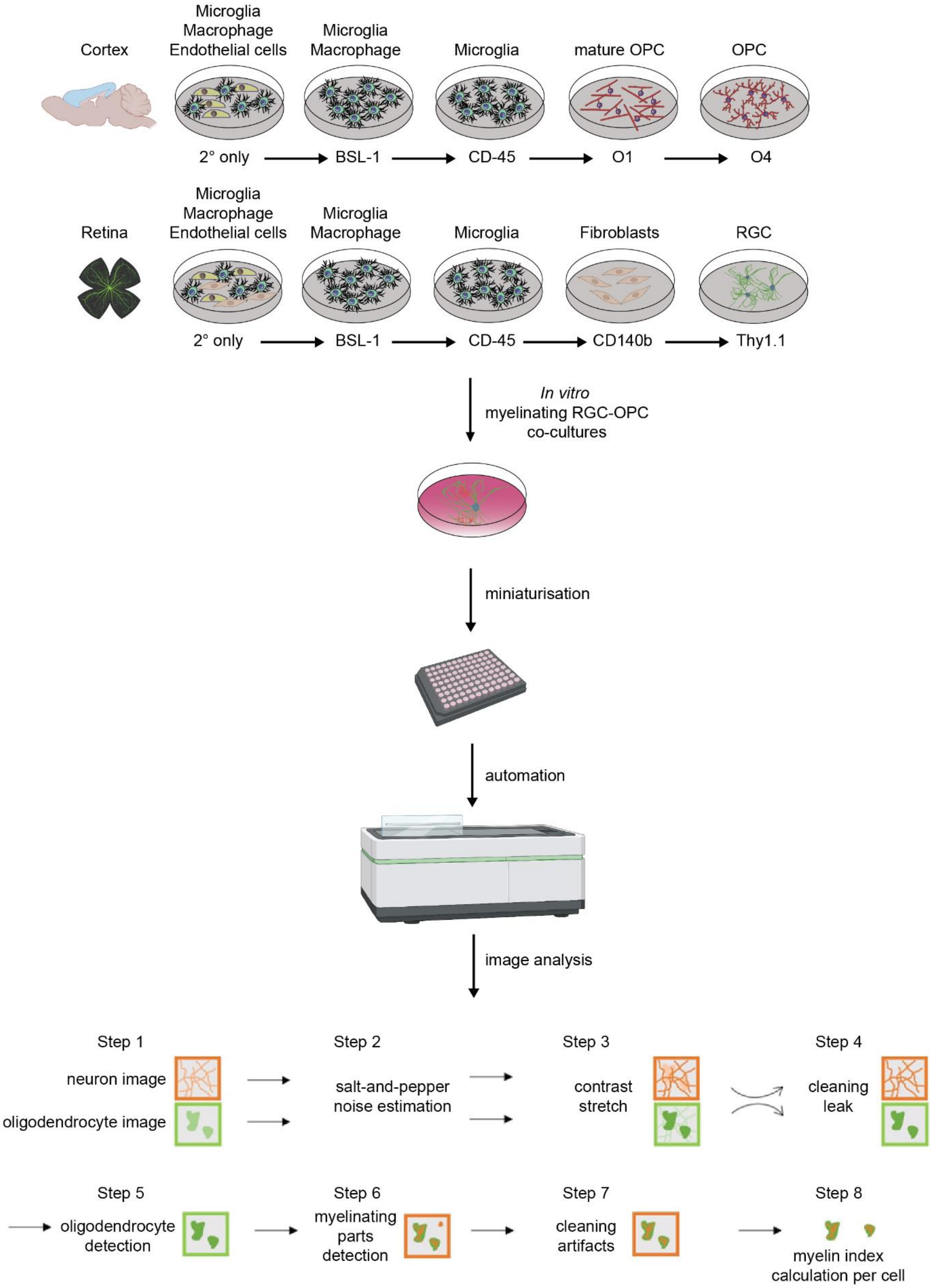

Supplementary figure 1 Experimental workflow

Overall experimental workflow of measuring and quantifying myelination *in vitro*, including the isolation of primary rat OPCs and RGCs by immunopanning, co-culturing of OPCs and RGCs and miniaturising the co-cultures into 96-well plates for higher throughput imaging with Perkin Elmer's Operetta/ Opera Phenix. Finally, the principle steps of image processing allowing myelination index calculation per OL cell. Briefly, neuron and OL images are pre-processed, cells are detected, myelination parts are detected and myelin index is calculated. Details for each step are found in Methods.

	Sigma Cat.	Final (µg/ml)
**Aqueous stock**		
L-Carnitine	C7518/C0283	2.0
Ethanolamine	E9508	1.0
D(+)-galactose	G0625	15
Putrescine	P5780	16.1
Sodium Selenite	S9133	0.01435
**Ethanolic stock**		
Corticosterone	C2505	0.02
Linoleic acid	L1012	1.0
Linolenic acid	L2376	1.0
Lipoic acid (thioctic acid)	T1395	0.047
Progesterone	P8783	0.0063
Retinyl acetate	R7882	0.1
Retinol, all trans (vit. A)	95144	0.1
D,L-alpha-Tocopherol (vit. E)	95240/T33251	1.0
D,L-alpha-Tocopherol acetate	T3001	1.0
**Add fresh**		
Albumin, bovine	A4161	2500
Catalase	C40	2.5
Glutathione (reduced)	G6013	1.0
Insulin	I6634	4.0
Superoxidase dismutase	S5395	2.5
Transferrin	T5391	5.0

Supplementary table 1 Vitamin B27 recipe
Ingredients of vitamin B27 stock solution
